# Preventing Tunnel Overlap in Revision Anterior Cruciate Ligament Reconstruction Through Creation of a Single Anatomic Oval Femoral Tunnel

**DOI:** 10.1016/j.eats.2025.103434

**Published:** 2025-01-22

**Authors:** Maureen Mae Acriche, Hiroshi Ohuchi, Takuya Okada, Shuzo Takazawa, Shin Yamada, Yuki Kato

**Affiliations:** Department of Sports Medicine, Kameda Medical Center, Kamogawa, Chiba, Japan

## Abstract

Femoral tunnel malposition is the main cause of failure in anterior cruciate ligament reconstruction. It not only contributes to the failure of initial anterior cruciate ligament reconstruction but is also of importance during the revision surgery. Tunnel positions can either be completely anatomic, completely nonanatomic, or partially anatomic. Cases with completely anatomic tunnels can make use of the same tunnel during the revision surgery, whereas cases with completely nonanatomic tunnels can make a new circular-shaped tunnel within the anatomic footprint. However, cases with partially anatomic tunnels raises concern as to where to create the new femoral tunnel within the anatomic footprint while avoiding overlap with the initial tunnel. By making the new femoral tunnel in an oval shape, we can prevent this from overlapping with the old partially anatomic tunnel while staying entirely within the anatomic footprint. The oval shape is achieved through an outside-in drilling technique using a FlipCutter and tilting the stepped drill sleeve. We present this surgical technique herein.

Anterior cruciate ligament (ACL) reconstruction is a common surgical procedure.^1^, ^2^, ^3^ Outcomes of this surgical intervention are successful; however, failure remains a problem. This is noted to be multifactorial and in terms of technical errors, femoral tunnel malposition is the main cause.^4^^,^^5^ This is when the femoral tunnel is created in a nonanatomic location and not in the native ACL footprint. Failure then results in revision surgery, which has 3 to 4 times the failure rate than primary ACL reconstruction.^6^ Multiple revision cases show that improper tunnel placement from the initial surgery still places the graft at risk for failure and, thus, should be corrected during revision.^7^

Regardless of primary or revision ACL reconstruction, reasons for failure boil down to tunnel malposition. In cases wherein the initial surgery resulted in a malpositioned location, which can be too anterior,^8^ creating the tunnel for the revision surgery now becomes vital. The most crucial decision for a surgeon is on how to make a new bone tunnel within the anatomic footprint with the presence of a previously created partial or nonanatomic bone tunnel while preventing the old and new tunnel from overlapping at the lateral wall of the intercondylar notch. The purpose of this Technical Note is to present a technique in making a new oval-shaped anatomic femoral tunnel during revision ACL reconstruction while preventing tunnel overlap with the old anteriorly located tunnel (Video 1).

## Surgical Technique

### Preoperative Imaging

**Fig 1:**
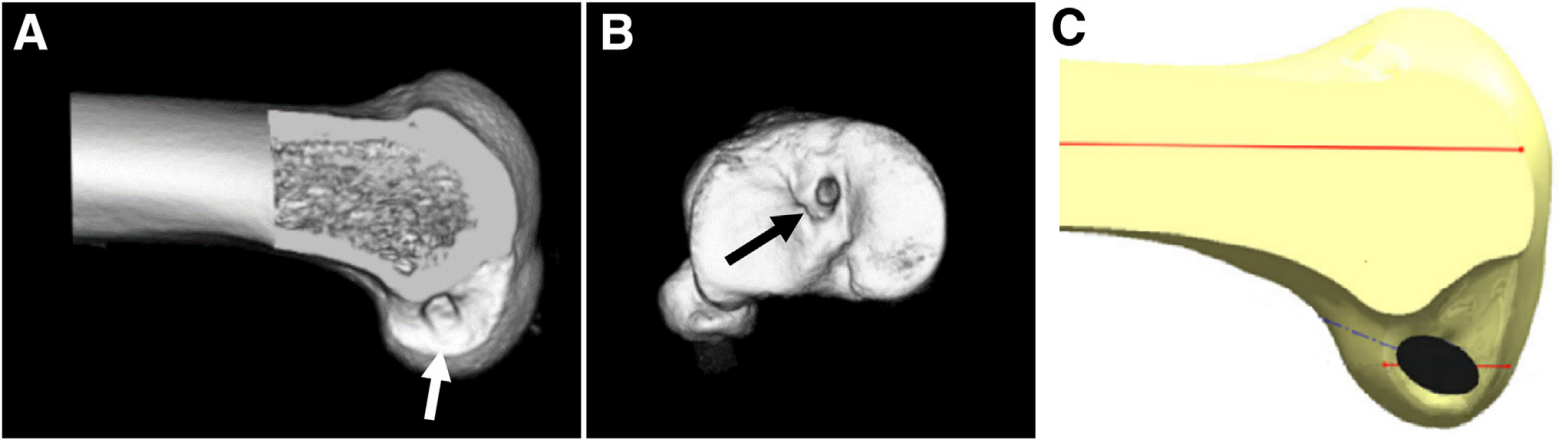
Bone tunnel from the initial surgery in comparison with the anatomic footprint. (A) 3D computed tomography scan of the patient’s left femur showing a circular-shaped femoral tunnel (white arrow) from the initial ACL reconstruction located too anterior. (B) 3D computed tomography scan of the patient’s left tibia showing the tibial tunnel (black arrow) from the initial ACL reconstruction in an anatomic location. (C) Anatomic location of the ACL femoral footprint (black oval) in a left femur. (Adapted from Lubowitz et al.^9^) (3D, 3-dimensional; ACL, anterior cruciate ligament.)

Preoperative imaging shows the bone tunnel from the initial surgery (Fig 1 A and B). The femoral tunnel is noted to be placed too anterior in comparison with the anatomic footprint as seen in Figure 1C.^9^

### Portal Placement

**Fig 2:**
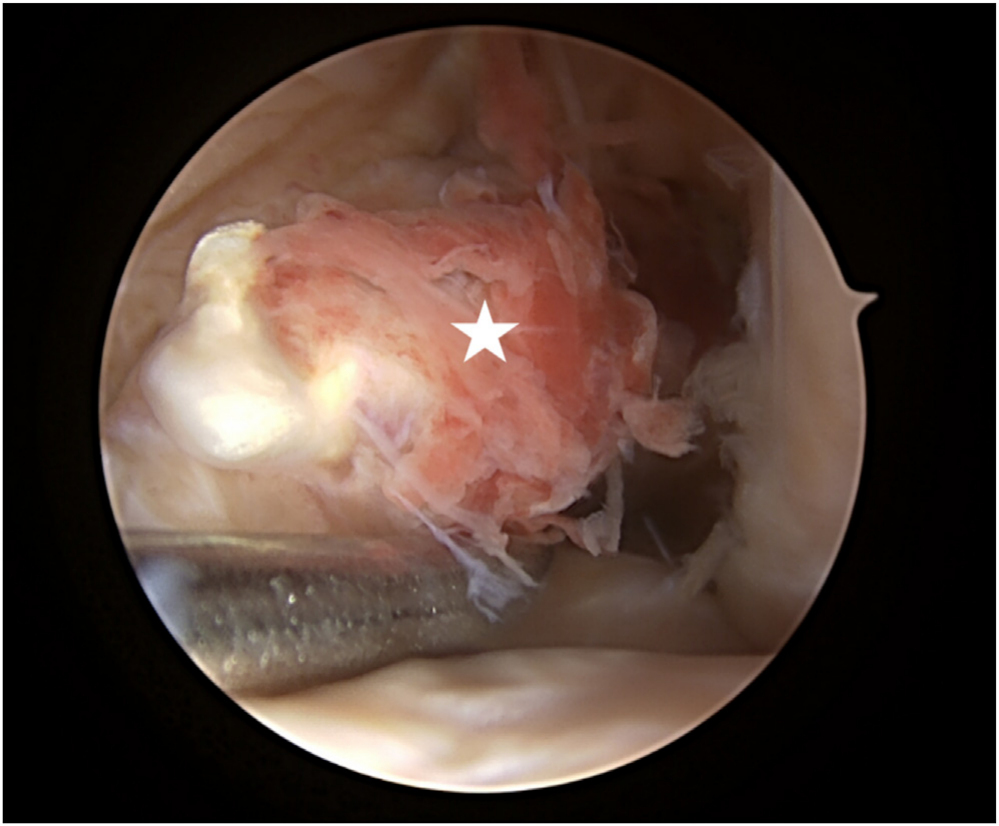
Intraoperative arthroscopic image of the previously reconstructed anterior cruciate ligament tendon (white star), which is torn proximally from the femoral attachment. Left knee viewed from the anterolateral portal.

Standard anterolateral portal and anteromedial portal is made. Diagnostic arthroscopy is performed revealing a rerupture of the previously reconstructed tendon (Fig 2).

### Graft Preparation

A 4-cm longitudinal incision is made medial to the tibial tuberosity to harvest the semitendinosus tendon. When the ipsilateral tendon was used during the initial ACL reconstruction, the contralateral semitendinosus tendon can be used. If there is difficulty in harvesting the semitendinosus tendon from the pes anserinus anteriorly, it can be done posteromedially from the popliteal fossa using an ultrasound-guided posteromedial tendon harvest technique.^10^ The graft is then prepared and quadrupled, with the free end whipstitched and the folded end passed through the loop of a TightRope RT (Arthrex Japan, Tokyo, Japan). The diameter is measured.

### Femoral Tunnel Preparation

**Fig 3:**
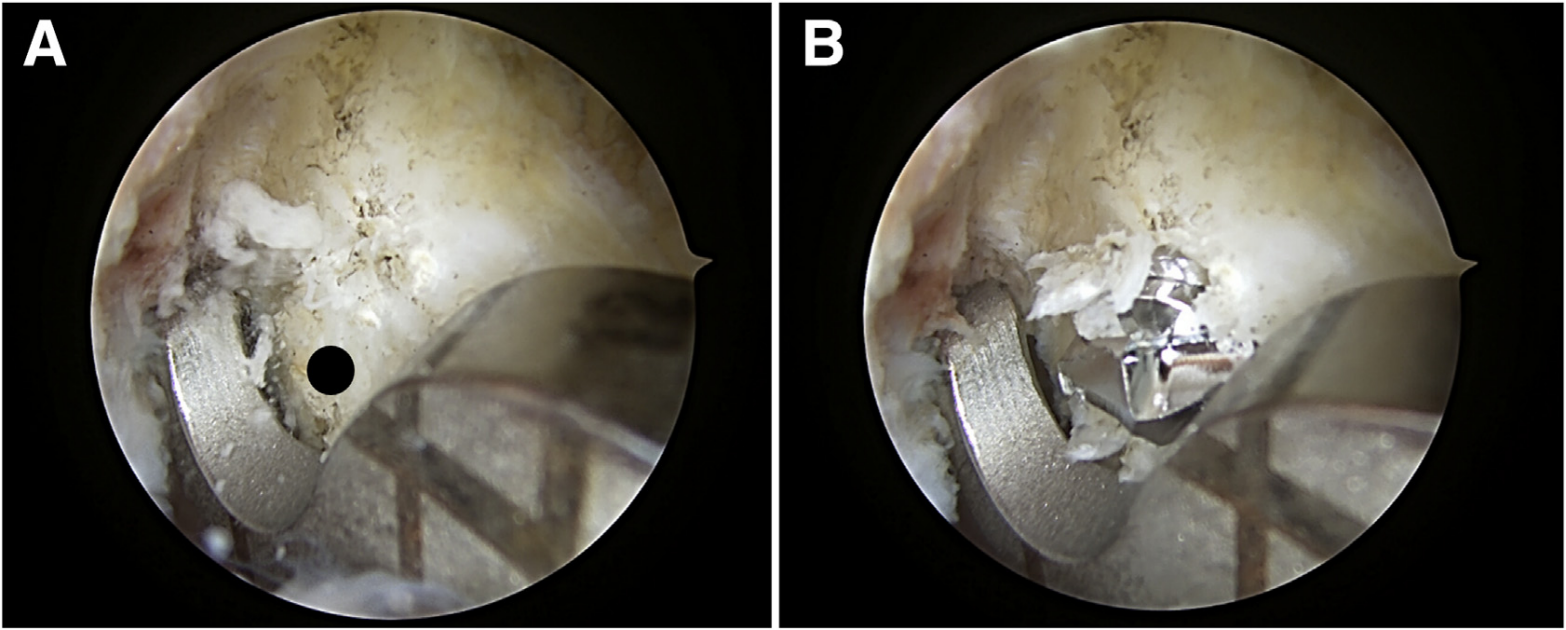
Placement of the new femoral tunnel. Left knee viewed from the anteromedial portal. (A) A footprint marking hook is used to mark the planned new bone tunnel (black circle). (B) A 3.5-mm FlipCutter exiting the lateral surface of the intercondylar notch.

**Fig 4:**
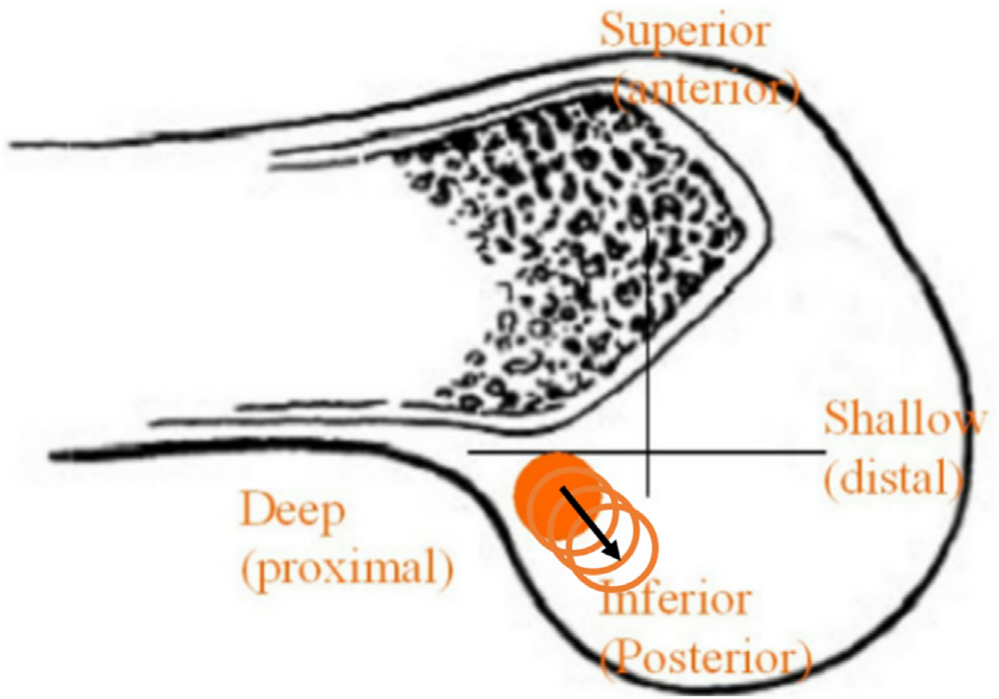
Orientation of new bone tunnel in a left femur. The solid orange circle represents the initial bone tunnel made prior to tilting the sleeve. The black arrow indicates the direction the tunnel is extended by tilting the sleeve. This is done to make an oval tunnel. (Adapted from Garofalo et al.^11^)

**Fig 5:**
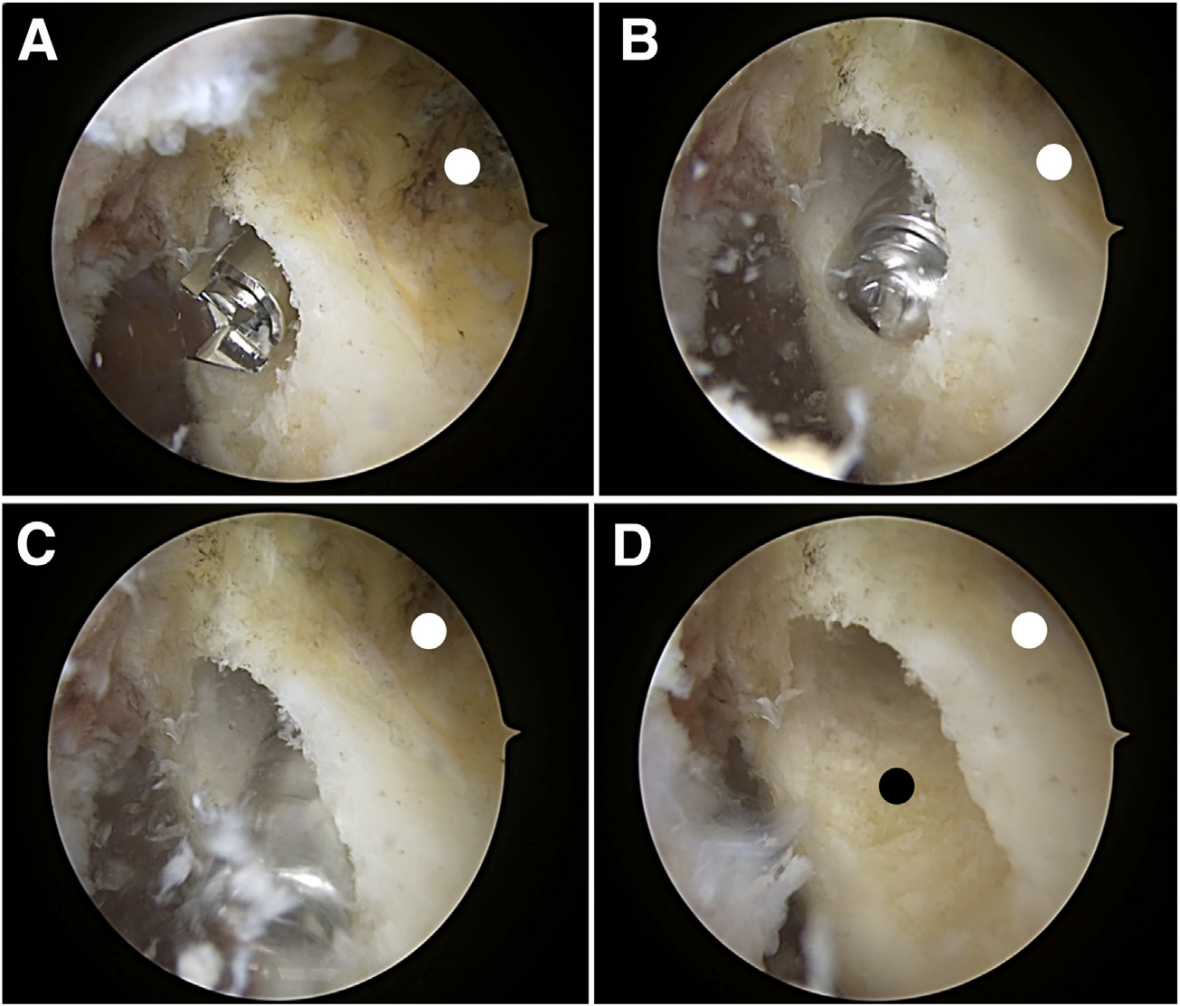
Creation of the new oval femoral tunnel using the FlipCutter. Left knee viewed from the anteromedial portal. The white circle indicates the anteriorly located tunnel from the initial surgery. (A) Initial drill hole is made using the FlipCutter. (B) The stepped drill sleeve is tilted so the drill tip points more inferior and distal while preventing tunnel overlap with the old anteriorly located tunnel. (C) Drilling of oval tunnel. (D) New oval femoral tunnel (black circle).

The knee is hung over the edge of the bed, maintaining the femur parallel to the floor. Any remnants from the torn ACL at the previous tunnel are debrided for a clearer view. Using the anteromedial portal as the viewing portal, the bone tunnel from the initial ACL reconstruction is visualized and the placement of the new bone tunnel is identified in terms of its location. The new tunnel is prepared by first marking the location using a radiofrequency device. A footprint marking hook (Arthrex Japan) is used to plan the placement of the new tunnel (Fig 3A). The drill sleeve is inserted 60° to a line perpendicular to the femoral anatomic axis and 20° to the transepicondylar axis, as described by Lubowitz et al.^9^ A 3.5-mm FlipCutter (Arthrex Japan) is then passed from the lateral cortex of the femur towards the lateral wall of the intercondylar notch ensuring that it is exiting at the right point (Fig 3B). Once inside the joint, the FlipCutter is opened and a larger diameter tunnel is reamed in a retrograde fashion. Then, to make the oval tunnel, the stepped drill sleeve (Arthrex Japan, Japan) is tilted so the drill points more inferior and distal (Fig 4).^11^ This is done with care so as not to pull the sleeve out from the femoral cortex completely (Fig 5 A-D).

### Determination of Tunnel Size

In creating the oval tunnel, we first measure the diameter of the graft bundle (*G*) and its corresponding graft cross-sectional area. For every *G* measured, the corresponding graft cross-sectional area is calculated using the following formula:

$$\text{Graft}\;\text{cross}-\text{sectional}\;\text{area}=\frac{\pi G^{2}}{4}$$

The corresponding cross-sectional area for each *G* is shown in Table 1.

**Table 1:** Graft Diameter (*G*) and the Corresponding Cross-Sectional Area $\left(\frac{\pi G^{2}}{4}\right)$

Graft Diameter = *G*	Graft Cross-Sectional Area = *πG*^*2*^*/4*
7	38.48451
7.5	44.17864668
8	50.26548245
8.5	56.74501729
9	63.61725122
9.5	70.88218423
10	78.53981633

The next step would be to determine which drill diameter (*D*) to use and how much to tilt it to achieve an appropriately sized oval tunnel. The drill is tilted in only one direction, and thus the minor axis of the oval tunnel is equivalent to *D*. For every major axis (*D′*), the corresponding oval tunnel cross-sectional area is calculated using the following formula:

$$\text{Oval}\;\text{tunnel}\;\text{cross}-\text{sectional}\;\text{area}=\frac{\pi DD^{\prime }}{4}$$

The corresponding oval tunnel cross-sectional area for each *D* and *D’* is shown in Table 2.

**Table 2:** Drill Diameter (*D*) and the Oval Tunnel Cross-Sectional Area $\left(\frac{\pi DD^{\prime }}{4}\right)$

Drill Diameter = *D*	Major Axis = *D'*	Minor Axis = *D*	Oval Tunnel Cross-Sectional Area = *πdd'/4*
5	10	5	39.269875
5	11	5	43.1968625
5	12	5	47.12385
5	13	5	51.0508375
5	14	5	54.977825
5	15	5	58.9048125
5	16	5	62.8318
6	9	6	42.411465
6	10	6	47.12385
6	11	6	51.836235
6	12	6	56.54862
6	13	6	61.261005
6	14	6	65.97339
6	15	6	70.685775
6	16	6	75.39816
6	17	6	80.110545

**Fig 6:**
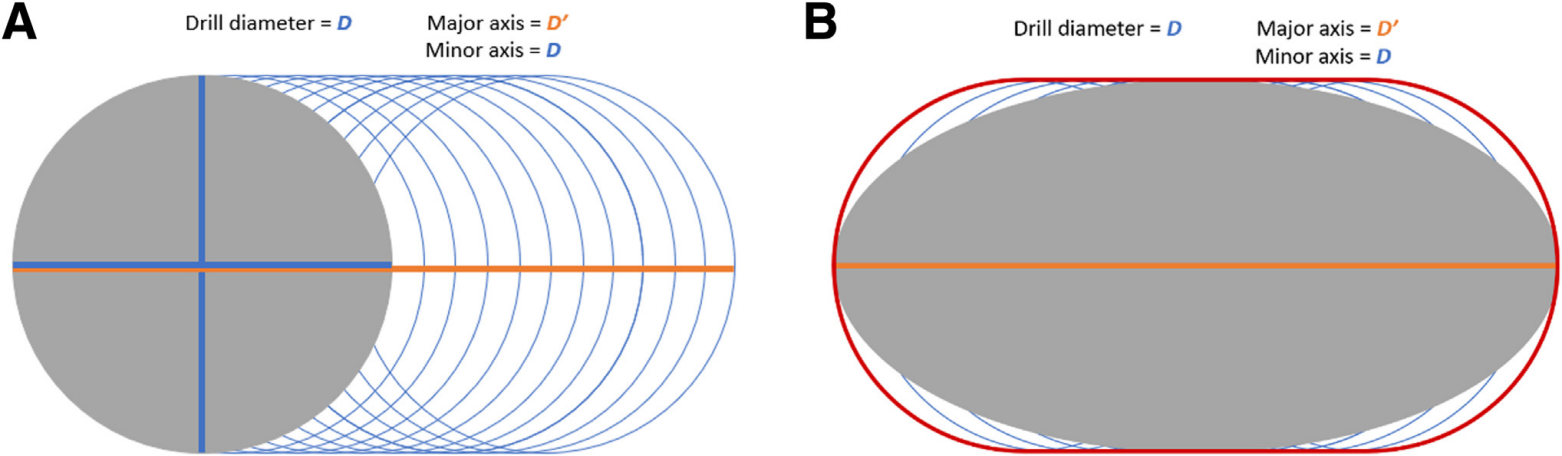
(A) Diagram showing the creation of an oval femoral tunnel. Once the graft size is identified, we choose the drill diameter (*D*) and the appropriate major axis (D′) of the oval tunnel. The drill is tilted in only one direction, and thus the minor axis of the oval tunnel is equivalent to *D*. The major axis is determined by how much the stepped drill sleeve is tilted. (B) The actual shape made after drilling the oval tunnel (encircled in red) can be approximated to an oval (filled in gray).

The data provided in Table 1 and 2 will serve as a guide in determining how much to tilt the drill in making the oval tunnel. Once we have measured our graft size, we now choose the drill diameter (*D*) and the appropriate major axis (D′) of the oval tunnel (Fig 6A). This is done by ensuring that the oval tunnel cross-sectional area is slightly larger than the graft cross-sectional area so that the graft would fit into the oval tunnel (Table 1). The actual shape produced upon moving the drill (encircled in red) is not a true oval. However, it can be approximated to the area of an oval (Fig 6B).

### Tibial Tunnel Preparation

In revision ACL reconstruction, the tibial tunnel often is reused because the initial tunnel is oftentimes placed anatomically. This is because for tibial tunnel creation, there is not much difficulty in visualizing the normal footprint.

### Graft Passage and Fixation

The graft is passed from the tibial tunnel upwards into the femoral tunnel with a TightRope RT (Arthrex Japan) attached on the folded end of the graft. When passing the graft through the oval-shaped femoral tunnel is difficult, a probe is used to turn and push the graft into the tunnel. The tibial side is fixed using a TightRope Attachable Button System (Arthrex Japan).

### Postoperative Imaging

**Fig 7:**
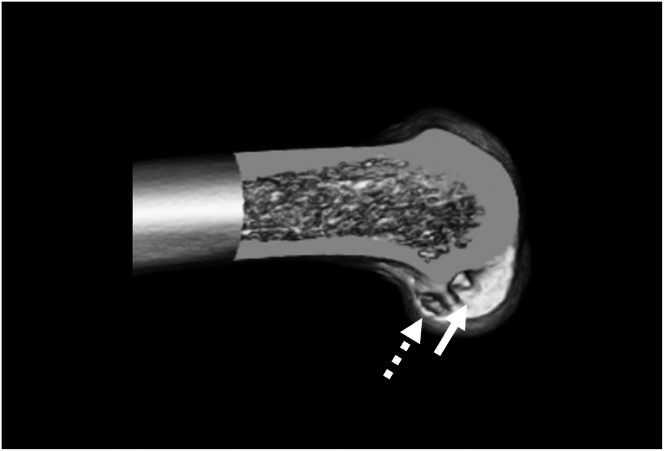
Postoperative 3-dimensional computed tomography scan of the patient’s left femur showing a new oval-shaped tunnel (white dashed arrow) deep and inferior to the initial tunnel (white solid arrow). The new tunnel is now located over the native anterior cruciate ligament footprint without tunnel overlap.

Postoperative imaging shows the new femoral oval tunnel located deep and inferior to the old tunnel (Fig 7). The tunnel is now positioned in an anatomic location while avoiding tunnel overlap.

## Discussion

ACL reconstruction using a conventional transtibial reconstruction technique usually results in an anteriorly oriented femoral tunnel because of limitation of movement of the drill in creating the femoral tunnel transtibially.^12^ A far medial portal technique also can result in an anteriorly located femoral tunnel in patients with limited knee flexion.^13^ These technical errors can lead to tunnel malposition, causing ACL rerupture and revision surgery.

Tunnel positioning plays a crucial part in revision ACL reconstruction. Tunnel locations can be divided into 3 categories: (A) completely anatomic tunnels that are completely within the anatomic footprint, (B) completely nonanatomic tunnels that are completely outside of the anatomic footprint, and (C) partially anatomic tunnels that are partially overlapping the anatomic footprint.^14^ Most revision cases have partially anatomic tunnels from the initial surgery, with space remaining. However, it would be difficult to create a circular tunnel because of the limited area. Thus, creating the new tunnel in an oval shape will enable passage of an adequately sized graft within the anatomic footprint while still preventing overlap with the previously created tunnel.

By applying our technique in using a FlipCutter (Arthrex Japan), we can clearly visualize the previous tunnel while creating an oval tunnel, since the drill will not be blocking the view, as is observed in an inside out technique. Thus, outside in drilling using this instrument enables reliable anatomic placement of the new tunnel (Table 3).^15^

**Table 3:** Advantages of This Outside-in Technique in Creating an Oval Tunnel

Enables clear visualization of previously created tunnel
Enables reliable anatomic placement of new tunnel
Avoids tunnel overlap with previously created tunnel

### Limitations

Our technique is only useful in cases whose initial femoral tunnel was placed partially anatomic. In completely anatomic tunnels from previous surgery, the same tunnel can be used during revision surgery, and in cases with completely nonanatomic tunnels, a new circular shaped tunnel can be made within the anatomic footprint. In addition, there is a learning curve in tilting the sleeve gradually in an ideal direction to create the oval tunnel without inadvertently pulling the sleeve out of the cortex (Table 4).

**Table 4:** Limitations of This Outside-in Technique in Creating an Oval Tunnel

Requires the use of a specific instrument
Technique only applicable to cases with partially anatomic tunnels
Learning curve necessary in tilting sleeve

## Disclosures

All authors (M.M.A., H.O., T.O., S.T., S.Y., Y.K.) declare that they have no known competing financial interests or personal relationships that could have appeared to influence the work reported in this paper.

## Supplementary Data

Video 1 This video shows our technique on preventing tunnel overlap in revision anterior cruciate ligament reconstruction through creation of a single anatomic oval femoral tunnel. The new femoral tunnel preparation is started by inserting a stepped drill sleeve 60° to a line perpendicular to the femoral anatomic axis and 20° to the transepicondylar axis. A 3.5-mm FlipCutter is then passed from the lateral cortex of the femur toward the lateral wall of the intercondylar notch and once inside the joint, this is opened and a larger diameter tunnel is reamed in a retrograde fashion. The stepped drill sleeve is then tilted to point more inferior and distal to make the oval tunnel. The most distinctive feature of our technique is that by making the new tunnel in an oval shape, overlap with the old tunnel is avoided while still creating an adequately sized tunnel within the anatomic footprint.
